# Supplementary material for: E-RespiNet: An LLM-ELECTRA driven triple-stream CNN with feature fusion for asthma classification
Source: PLoS One. 2025 Nov 4;20(11):e0334528. doi: 10.1371/journal.pone.0334528 (PMC12585061; doi:10.1371/journal.pone.0334528)
Supplement: Attachment — Submitted filename: PONE-D-25-28806 Review.docx [file pone.0334528.s001.docx]

Here are several high‑impact grounds on which a reviewer could recommend outright rejection of this manuscript: PONE-D-25-28806

1. **Insufficient Novelty**
   - The core idea—multi‑stream CNNs on MFCCs, wavelets and mel‑spectrograms—has been explored extensively in prior respiratory‑sound studies (e.g., Wang et al. 2022; Lee et al. 2023). The authors’ adaptation of ELECTRA adds only a marginal ~2% gain and does not constitute a clear conceptual advance .
2. **Questionable Generalization and Overfitting**
   - A 25.5% drop in accuracy under cross‑domain testing signals severe overfitting to the training sites. This level of performance degradation far exceeds acceptable thresholds for clinical tools and suggests the model does not truly capture disease‑relevant features .
3. **Lack of Statistical Rigor**
   - No confidence intervals, p‑values or hypothesis tests are reported for any comparisons. Without measures of variance or significance testing, the claimed “98.3% accuracy” cannot be trusted as anything beyond dataset‑specific chance performance .
4. **Opaque Methodological Reporting**
   - Critical details (e.g. random seed settings, exact train/validation splits, hardware specs, hyperparameter search ranges) are missing. This violates basic reproducibility standards for machine‑learning studies and precludes any possibility of replication .
5. **No Clinical or Ethical Oversight**
   - There is no mention of IRB approval, participant consent or real‑world deployment constraints. For a paper claiming medical‑diagnostic utility, this omission is a fundamental breach of publication ethics .
6. **Excessive Model Complexity with Unrealistic Deployment Path**
   - A 119 M‑parameter architecture requiring multiple augmentation steps and high‑end GPUs is impractical for most clinical settings, especially in low‑resource environments. No discussion of model pruning, latency or power requirements is provided .

**Recommendation to Reject**

Given the combination of marginal novelty, overfitting, lack of statistical validation, irreproducibility, and ethical omissions, this manuscript falls well below the standards for a publishable, clinically relevant study. I therefore recommend outright rejection.
