# Supplementary material for: E-RespiNet: An LLM-ELECTRA driven triple-stream CNN with feature fusion for asthma classification
Source: PLoS One. 2025 Nov 4;20(11):e0334528. doi: 10.1371/journal.pone.0334528 (PMC12585061; doi:10.1371/journal.pone.0334528)
Supplement: Attachment — Submitted filename: Review Comments for Manuscript.docx [file pone.0334528.s002.docx]

**Review Comments for Manuscript ID: PONE-D-25-28806**

The manuscript is technically promising with strong experimental results. But model validation, generalization , and reproducibility are of concern. A more concise presentation and improvements in visual quality will also enhance the overall impact. The authors may wish to address this in their revisions of the manuscript.

1. **Novelty and Justification for ELECTRA Integration**

Although the use of ELECTRA in an audio-based diagnostic model is novel, the paper does not present compelling arguments for choosing it over other audio-specific transformer models like Wav2Vec 2.0 or AST. In the absence of comparison or ablation studies, the advantages of ELECTRA are still a claim.

2. **Practical Limitations and Deployment Feasibility**

The processor-heavy nature of the proposed model (ELECTRA with 12 transformer layers and three parallel CNNs) puts into question the feasibility of deploying it to low-resource settings. There is no indication in the manuscript of inference time, memory footprint, or hardware requirements to understand how this work would be applicable to the environments they propose are possible.

3. **Writing Redundancy and Overstatements**

The manuscript tends to make the same points over and over, such as the novelty of the integration of ELECTRA and the three-stream CNN. Terms such as, “first adaptation,” or “new paradigm” are applied without appropriate substantiation. A more muted and neutral tone would be more clear and credible.

4. **Low Quality of Figures**

The resolution of Figures 2 and 4 is poor. The text is difficult to read, and visual details are blurred. Authors should replace these with high-resolution vector graphics to ensure clarity for readers and reviewers.

5. **Explainability and Visualization**

Although the Grad-CAM is referenced as interpretability technique, there is no explicit clear visual depiction or discussion of the way in which the Grad-CAM outputs correlate with clinical decisions. This undermines the explainability of AI. It would also be useful to include visualizations and interpretations from experts on sample.

6. **Inconsistent Formatting and Spacing Throughout the Manuscript**

Throughout the manuscript, there are several inconsistencies in formatting, including irregular line spacing, mismatched fonts, inconsistent use of equation formatting, and uneven paragraph alignment. These issues make the paper difficult to read and professionally review. A thorough formatting revision is necessary to ensure clarity, readability, and adherence to the journal’s submission standards.
